# Contradictory and weak evidence on the effectiveness of anti-emetics for MTX-intolerance in JIA-patients

**DOI:** 10.1186/s12969-018-0229-x

**Published:** 2018-02-15

**Authors:** Casper G. Schoemaker, E. H. Pieter van Dijkhuizen, Sebastiaan J. Vastert

**Affiliations:** 1 Department of Pediatric Rheumatology and Immunology, Wilhelmina Children’s Hospital, University Medical Center Utrecht, Room KC.03.063.0, P.O. box 85090, 3508 AB Utrecht, Netherlands; 2 Netherlands JIA Patient and Parent Organization, Member of ENCA, Rijen, The Netherlands; 3 National Institute for Public Health and the Environment (RIVM), Bilthoven, The Netherlands; 4 Faculty of Medicine, Utrecht University, Utrecht, Netherlands

**Keywords:** Juvenile Idiopathic Arthritis (JIA), Methotrexate, MTX-intolerance, Anti-emetics, Co-medication

Methotrexate (MTX) is an effective, relatively safe, and low-cost treatment for children with JIA, but its use is often limited by the occurrence of so called intolerance in a significant number of patients [1, 2]. Many JIA patients receive anti-emetics as a co-medication to prevent or treat MTX-intolerance. In a recent review in this journal Falvey et al. concluded: “Co-medication with anti-emetics, such as ondansetron, appears to be a highly effective approach” [2]. In our view this firm conclusion was based on weak and indirect evidence. Here, we aim to put into perspective both the timing of the outcome measures and the population size of the studies Falvey referred to.

To underpin their conclusion Falvey et al. referred essentially to two studies (references 27 and 28) [2]. Kempinska et al. performed a Randomized Controlled Trial (RCT) in patients with Crohn’s disease. The outcomes were measured 3 months after the start of MTX. From other studies we know that for most JIA patients MTX-intolerance rarely starts before 4 months [1, 3]. Blanco et al. performed an observational study in 9 adult rheumatoid arthritis (RA) patients. It was a very small study, lacking a control group. The other papers on ondansetron Falvey et al. referred to (their references 18, 24–26) were not relevant for MTX-intolerance, as the authors already stated [2].

Recently two large studies on anti-emetics for MTX-intolerance in pediatric patients have been published, providing some evidence against the conclusion by Falvey et al. [4, 5]. Scheuern et al. described the results of a prospective, observational study in 196 JIA patients. They concluded that “various modalities used as countermeasures against nausea (…) showed no discernible effect” [4]. Dupont-Lucas et al. performed an observational study in 102 pediatric IBD patients. They concluded: “Prophylactic prescription of anti-emetics (…) did not prevent symptoms of MTX intolerance” [5]. Both studies were not randomized, so firm conclusions cannot be drawn from these studies either.

In conclusion: thus far, there is only weak and contradictory evidence on the effectiveness of the (prophylactic) use of anti-emetics for prevention or treatment of MTX-intolerance in JIA. We will need a large, double blind RCT in JIA patients on the prophylactic use of anti-emetics to establish its effectiveness in preventing intolerance. In our view this is a very relevant research question for JIA patients, parents and clinicians.
